# Flow cytometric analysis reveals the high levels of platelet activation parameters in circulation of multiple sclerosis patients

**DOI:** 10.1007/s11010-017-2955-7

**Published:** 2017-02-16

**Authors:** Agnieszka Morel, Joanna Rywaniak, Michał Bijak, Elżbieta Miller, Marta Niwald, Joanna Saluk

**Affiliations:** 1 Department of General Biochemistry, Faculty of Biology and Environmental Protection, University of Lodz, Pomorska 141/143, 90-236 Lodz, Poland; 2 Department of Physical Medicine, Medical University of Lodz, Pl. Hallera 1, 90–647 Lodz, Poland; 3 Neurorehabilitation Ward, III General Hospital in Lodz, Milionowa 14, 93–113 Lodz, Poland

**Keywords:** Multiple sclerosis, Blood platelets, Flow cytometry, Prothrombotic risk

## Abstract

The epidemiological studies confirm an increased risk of cardiovascular disease in multiple sclerosis, especially prothrombotic events directly associated with abnormal platelet activity. The aim of our study was to investigate the level of blood platelet activation in the circulation of patients with chronic phase of multiple sclerosis (SP MS) and their reactivity in response to typical platelets’ physiological agonists. We examined 85 SP MS patients diagnosed according to the revised McDonald’s criteria and 50 healthy volunteers as a control group. The platelet activation and reactivity were assessed using flow cytometry analysis of the following: P-selectin expression (CD62P), activation of GP IIb/IIIa complex (PAC-1 binding), and formation of platelet microparticles (PMPs) and platelet aggregates (PA) in agonist-stimulated (ADP, collagen) and unstimulated whole blood samples. Furthermore, we measured the level of soluble P-selectin (sP-selectin) in plasma using ELISA method, to evaluate the in vivo level of platelet activation, both in healthy and SP MS subjects. We found a statistically significant increase in P-selectin expression, GP IIb/IIIa activation, and formation of PMPs and PA, as well as in unstimulated and agonist-stimulated (ADP, collagen) platelets in whole blood samples from patients with SP MS in comparison to the control group. We also determined the higher sP-selectin level in plasma of SP MS subjects than in the control group. Based on the obtained results, we might conclude that during the course of SP MS platelets are chronically activated and display hyperreactivity to physiological agonists, such as ADP or collagen.

## Introduction

Multiple sclerosis (MS) is a chronic, demyelinating immune-mediated disease of the central nervous system (CNS) with axonal degeneration and astrogliosis [1]. There are three main clinical types of MS, defined as relapsing-remitting (RR), secondary progressive (SP), and primary progressive (PP) with a progressive-relapsing (PR) subtype [2]. It is generally very difficult to predict the clinical course of MS [3]. Histopathologically, MS is considered as a heterogenous neurological disease with a variable clinical course and several pathophysiological mechanisms, such as focal inflammatory infiltrates, demyelination, gliosis, axonal/neuronal damage within the CNS, and in some cases remyelination and repair mechanisms [4, 5]. A great number of patients with the most common type RR MS gradually develop neurological deterioration independent of relapses. When axons lose their ability to regenerate, RR MS patients develop SP MS dominated by neuroaxonal degeneration. The axonal loss is responsible for the transition from RR MS to progressive types of the disease. In SP MS, the permanent clinical disability correlates with axonal injury as well brain and spinal cord atrophy [6–10] in SP MS and in RR MS active tissue injury is associated with inflammation but in SP MS the inflammatory response occurs at least partly behind the blood–brain barrier (BBB), which makes it more difficult to treat [11]. In MS, the immune cells, mainly autoreactive T cells (CD4+, CD8+), infiltrate the CNS and release proinflammatory cytokines that activate phagocytic cells (macrophages), leading to the inflammation of white matter and subsequent myelin destruction [5, 12].

The blood platelets possess a large variety of compounds stored in α-granules, numerous membrane receptors, immunomodulatory mediators, and cell adhesion molecules, through which they affect the permeability of BBB. Blood platelets are responsible for the infiltration of autoreactive T cells which form new neuroinflammatory lesions in CNS [13–15]. Due to neuroinflammation disorders of the BBB, platelets rapidly adhere to the endothelium cells, became activated, and contribute to neurovascular inflammation caused by multiple interactions with endothelial and inflammatory cells [16, 17]. Therefore, in the inflamed neuronal tissue platelets contribute to neurovascular inflammation and neuronal disease in several ways [18, 19]. Platelets may modulate inflammation by their receptor-dependent interaction with leukocytes and the release of bioactive mediators, mainly matrix metalloproteinases and different chemokines, and it is one of the most important mechanisms since MS is mediated via migration of leukocytes and monocytes from the peripheral circulation to CNS [20]. Under inflammatory conditions, stimulated platelets rapidly adhere to the endothelium or to the subendothelial extracellular matrix at sites of vascular endothelial injury [14, 21–23]. The platelets are also a major source of IL-1α which can activate brain endothelium and enable the entry of white blood cells leading to cerebrovascular inflammation [24].

It has been proved that blood platelets might be a key player in the development of inflammation in early stage of MS [23]. RR MS and SP MS are two different types of MS characterized by different ongoing pathogenic processes. RR MS is characterized mainly by inflammation processes, while SP MS (the chronic progressive stage) is characterized with the domination of neurodegenerative processes. The clinical symptoms are also different. Currently, many studies targeted one type of MS to make their study more specific [25, 26]. The most important is to know the differences between these two basic types in this heterogenic, complex disease.

The platelet activation and the role of platelets in hemostatic mechanisms in a further development of the disease in the secondary progressive stage are the subject of our intensive studies for some years. Our previous findings suggest that platelet activation may be an epiphenomenon consequent to the disease processes in MS, probably secondary to endothelial injury, which causes the exposure of platelets to a variety of stimuli. We have demonstrated in vitro that isolated platelets obtained from patients with SP MS have adhered significantly stronger to typical subendothelial thrombogenic proteins—collagen and fibrinogen—than platelets from healthy controls [27]. Furthermore, we have shown an increased platelet aggregation in SP MS measured in platelet-rich plasma (PRP) due to ADP, collagen, or arachidonate action [27, 28].

In the current study, we used flow cytometry, a sensitive method routinely used both in the diagnosis of health disorders, as well as in basic research. The platelet subpopulations (normal platelets, microparticles (PMPs), and platelet aggregates (PA) in whole blood were distinguished from other cells based on the presence of constitutive platelets’ membrane receptor (CD61) on their surface and light-scattering properties of platelets, which are linked to cell size and granularity. Additionally, the use of different antigen-specific and fluorophore-conjugated primary antibodies allowed the quantitative determination of target antigen expression on the cells. In our experiments, we measured the level of expression of platelet activation marker CD62P (P-selectin) and the activation of integrin receptor GP IIb/IIIa. Using flow cytometry technique, it was possible to analyze the biological activity of platelets in their natural environment, immediately after blood collection. Therefore, the obtained results, for the first time, allowed us to directly understand the degree of activation and reactivity of platelets in the circulation of patients with SP MS. All studies were performed without and with exposure to the physiological agonists: ADP and collagen.

In addition, we studied the level of the soluble adhesion molecule sP-selectin (soluble P-selectin) in plasma from patients with SP MS, as a biomarker of inflammation and platelet activation in vivo.

## Materials and methods

### Materials

Flow cytometry reagents such as anti-CD61/PerCP, anti-CD61/PE, anti-CD61/FITC, anti-CD62/PE, PAC-1/FITC, and Cellfix were obtained from Becton Dickinson (San Diego, CA, USA). ADP and collagen were purchased in Chrono-Log (Havertown, PA, USA). All other chemicals were of analytical grade or highest quality available products. Human sP-selectin ELISA kit (Invitrogen).

### Patients and samples

The human blood samples were collected into CPDA-1 (citrate phosphate dextrose adenine-1) bags from 85 patients suffering from secondary progressive (SP) course of MS. Patients were observed for one year before and diagnosed according to the revised McDonald’s criteria. SP MS was ascertained as defined by Lubin et al. [29]. The SP MS can be recognized when initial relapsing-remitting course is followed by progression, with or without occasional relapses, minor remissions, and plateaux. Clinical characteristics of SP MS patients are as follows: mean age 48.6 ± 12.5 years, BMI 21.1 ± 9.7, disability status scale (EDSS) 4.5 ± 1.8, and mean disease duration 12.3 ± 9.5 years. Blood samples were delivered from Neurological Rehabilitation Division III General Hospital in Lodz, Poland. Platelet counts of SP MS patients were measured in the clinical laboratories of the hospital. Normal ranges of values were used for reference. Mean platelet counts for both patients (*n* = 85) and control (*n* = 50) were within the normal range (150–400 × 10^3^/μL). Patients did not receive any medications modifying platelet function.

Human blood samples obtained from healthy volunteers were a control (*n* = 50). Healthy volunteers were not taking any medications, and they have never been diagnosed with MS or other chronic disease, any neurological or hormonal illness, and any chronic inflammatory disease. Both groups (patients and control) were matched by age and sex.

Fifty two female patients with SP MS and 31 healthy female subjects were considered for the study. None of these women were undergoing hormone replacement therapy.

All blood samples (from patients and control group) were collected in the morning (between 8 am and 10 am) in fasting status and stored using the same protocol. The protocol and all procedures were done according to the Helsinki Declaration and were approved by the Ethics Committee of the Medical University of Lodz, Poland, RNN/260/08/KB, and the Ethics Committee of the Faculty of Biology and Environmental Protection of University of Lodz, Poland, No. 5/KBBN-UŁ/II/2013.

### Flow cytometry analysis

The changes in the activation and reactivity of resting or agonist-stimulated blood platelets were assessed by three-color flow cytometry. The fresh whole blood samples, without agonist or activated with ADP (20 µM, 10 min., 37 °C) or collagen (20 µg/ml, 10 min., 37 °C), were fixed in 1% Cellfix solution (9:1) for 1h at 37 °C and stained with anti-CD61/PerCP, anti-CD62P/PE, and PAC-1/FITC antibodies (30 min in dark, RT). Before analysis, the prepared samples were dissolved in 0.9% NaCl (20:1) and vortexed. Fluorescence of 10,000 platelets (CD61/PerCP-positive objects) was measured with an LSR II Flow Cytometer (Becton Dickinson, San Diego, CA, USA). The platelets were distinguished from other cells based on forward light scatter (FSC) *vs*. side light scatter (SSC) plot characteristics on a log/log scale (first gate) and by positive staining with monoclonal antibody CD61/PerCP (second gate). The percentages of CD62P-positive platelets and PAC-1-positive platelets were calculated relative to the total number of platelets (CD61-positive cells) present in each sample. Antibody-positive platelets were defined as exhibiting fluorescence intensities greater than 99% of unstained samples. To compensate for non-specific immunofluorescence, the percentages of CD62P- and PAC-1-positive platelets were obtained after subtracting the percentage of positive platelets when the antibodies were replaced by the isotype-matched PE- and FITC-conjugated immunoglobulin controls for CD62P (isotype IgG1) and PAC-1 (isotype IgM), respectively.

Additionally, the fractions of platelet-derived microparticles (PMPs) and platelet aggregates (PA) in unstimulated or agonist-activated whole blood samples were measured in the experiments of P-selectin expression and PAC-1 binding to platelets. Platelet microparticles and platelet–platelet or platelet–leukocyte aggregates were distinguished from platelets (CD61-positive objects) based on their size and granularity on the forward light scatter (FSC) *vs*. side light scatter (SSC) plots. All data analyses were performed in FACSDiva version 6.1.2.

### sP-selectin measurement

In plasma obtained from patients with SP MS and healthy volunteers, the concentration of sP-selectin was determined by immunosorbent method (ELISA). The level of sP-selectin in human plasma was measured using P-selectin (soluble) Human ELISA Kit (Invitrogen) according to the manufacturer’s protocol. The level of sP-selectin in plasma was determined from a standard curve expressed as ng/mL.

### Statistical analysis

Data were statistically elaborated. The expression levels of platelet activation and aggregation biomarkers are presented as mean value ± SD. The Shapiro–Wilk test was used to verify whether the data were normally distributed. The significance of differences between samples and controls was determined with unpaired Student’s *t* or Mann–Whitney *U* test. The data were analyzed using StatsDirect statistical software V. 2.7.2. The value of *p* < 0.05 was considered as statistically significant.

## Results

To measure the activation of platelets and their reactivity to physiological agonists (ADP and collagen) in whole blood obtained from SP MS patients or healthy controls, we performed three-color flow cytometry as an investigation method. Based on flow cytometry analysis, our results clearly indicated the statistically significant increase in both basal and agonist-treated platelet activation states in patients with SP MS compared with healthy individuals.

Using forward light scatter (FSC) vs. side light scatter (SSC) plot characteristics, we determined in CD61/PerCP-positive objects the formation of platelet aggregates as well as the release of platelet-derived microparticles. Using reference beads, we estimated gates: CD61/PerCP-positive objects with FSC lower than 10^2.3^ were characterized as PMPs, while objects with FSC higher than 10^4^ were characterized as PA. In samples of non-stimulated platelets, we observed the augmented basal level of PA (2.3-fold vs. control; p < 0.05) (Fig. 3a). Moreover, we showed the increased pool of PMPs (2.5-fold vs. control; *p* < 0.01) observed in FSC vs. SSC cytometry dot plots (Fig. 4a). We chose the representative dot plots of percentage of PA and PMPs: for non-stimulated control, 1.8% PA and 1.6% PMPs (Fig. 7a) and for non-stimulated SP MS, 4.9% PA and 3.6% PMPs (Fig. 7d). The values of the PA and PMPs was respectively 2.7 times and 2.5 times higher in SP MS than in control.

**Fig. 1:**
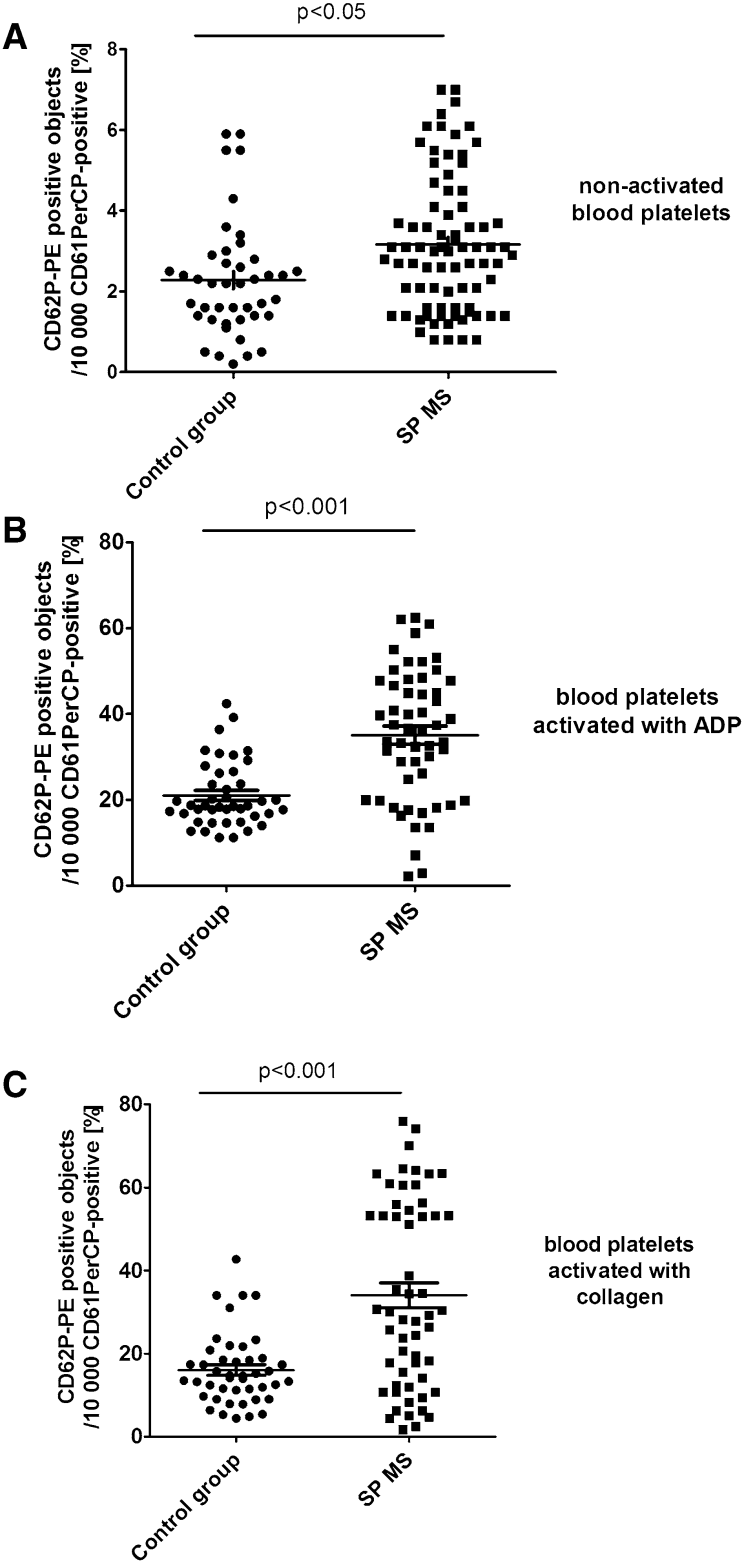
Expression of P-selectin on resting (**a**) or agonist-stimulated platelets: ADP 20 µM (**b**) and collagen 20 µg/ml (**c**) in whole blood samples obtained from SP MS patients and healthy controls. Analyses were performed in whole blood of healthy volunteers (control) and patients with SP MS. The blood platelets were distinguished based on the expression of CD61/PerCP. For each sample, 10 000 CD61-positive objects (platelets) were acquired. For the assessment of P-selectin expression, samples were labeled with fluorescently conjugated monoclonal antibody CD62P/PE. Results are shown as the percentage of platelets expressing CD62P. Statistical analysis was performed using Mann–Whitney *U* test

**Fig. 2:**
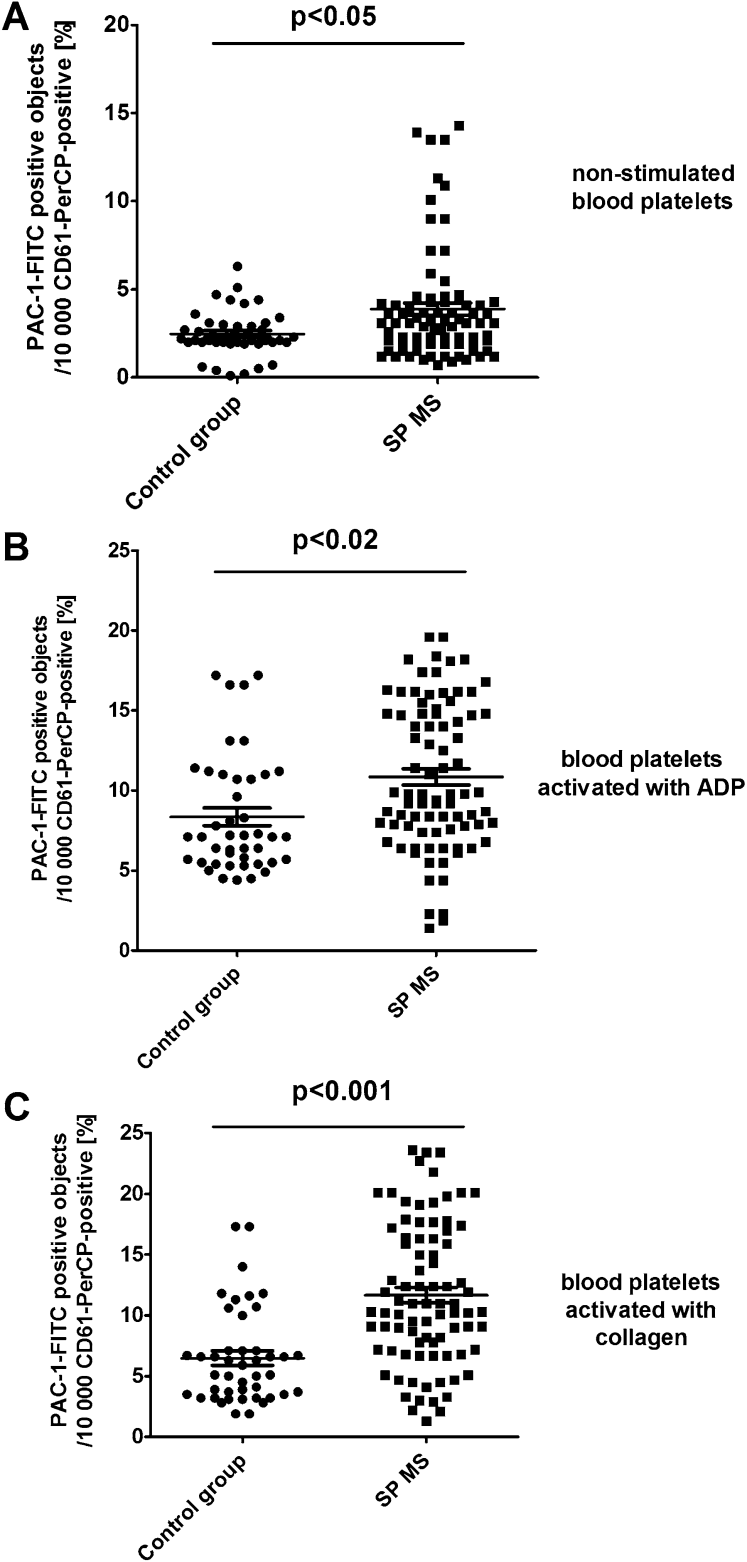
Expression of the active form of GPIIb/IIIa on resting (**a**) or agonist-stimulated platelets: ADP 20 µM (**b**) and collagen 20 µg/ml (**c**) in whole blood samples obtained from SP MS patients and healthy controls. Analyses were performed in whole blood of healthy volunteers (control) and patients with SP MS. The blood platelets were distinguished based on the expression of CD61. For each sample, 10,000 CD61-positive objects (platelets) were acquired. For the assessment of P-selectin expression, samples were labeled with fluorescently conjugated monoclonal antibody PAC-1-FITC. Results are shown as the percentage of platelets binding PAC-1/FITC. Statistical analysis was performed using Mann–Whitney *U* test

Additionally, in CD61/PerCP-positive objects PE and FITC fluorescence was detected. Gates were estimated based on unstained probes. In case of PE, the objects with fluorescence level higher than 10^2.7^ were characterized as platelets with surface expression of P-selectin, while in the case of FITC the objects with level of fluorescence higher than 10^3.05^ were characterized as platelets with PAC-1 antibody binding. The percentages of CD62P-positive platelets and PAC-1-positive platelets were calculated relative to the total number of platelets (CD61-positive cells) present in each sample. The level of basal platelet activation was measured by the surface expression of two main platelet markers: CD62P and PAC-1 binding to activated form of GPIIb/IIIa. The resting platelets in whole blood samples obtained from SP MS patients showed 2.3-fold higher CD62P expression (p < 0.05) (Fig. 1a) and 1.5-fold higher level of PAC-1 binding to activated form of GPIIb/IIIa(p < 0.05) (Fig. 2a) in comparison to the control group. Figure 5 shows the representative histograms of the percentage of CD62P-positive platelets in samples from non-stimulated healthy control, which is 1.9% (A), and non-stimulated blood of SP MS patients, which is 5.9% (D). The histograms of the percentage of PAC-1-positive platelets for the same analyzed subjects are shown in Fig. 6. The ratio between healthy non-stimulated control (1.7%) (Fig. 6a) and non-stimulated SP MS platelets (4.3%) (Fig. 6d) is 2.5.

**Fig. 3:**
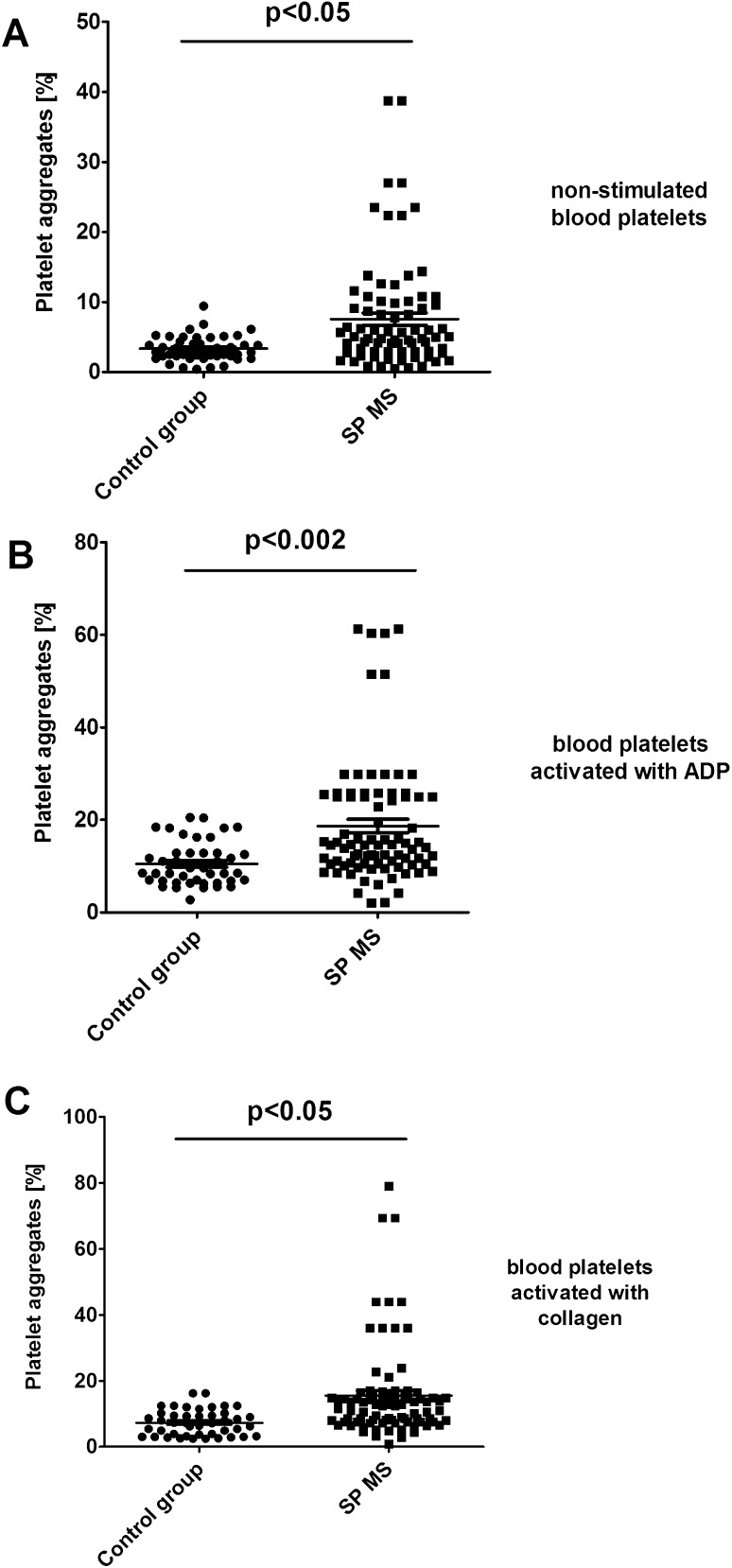
Formation of platelet aggregates in control (**a**) and agonist-stimulated platelets: ADP 20 µM (**b**) and collagen 20 µg/ml (**c**) in whole blood samples obtained from SP MS patients and healthy controls. The fraction of platelet aggregates was distinguished from platelets (CD61-positive objects) based on their size and granularity on the forward light scatter (FSC) vs. side light scatter (SSC) plots. CD61-positive objects with FSC higher than 10^4^ were characterized as platelet aggregates. In each sample, 10,000 CD61-positive objects (platelets) were measured. Statistical analysis was performed using Mann–Whitney *U* test

In our study, we also compared the level of blood platelet responsiveness to two physiological agonists. The activation of platelets in whole blood treated with ADP (20 µM) was statistically significantly higher in SP MS patients compared to the control group, which we observed in the expression of CD62P (over 60%;* p* < 0.001) (Fig. 1b) and PAC-1 binding (over 30%;* p* < 0.02) (Fig. 2b). However, according to the representative histograms (Figs. 5, 6), the difference between a randomly selected healthy control and SP MS subject may be much greater. The percentage of CD62P-positive platelets for healthy controls was 21.2% (Fig. 5b), and for SP MS sample it was 37.5% (Fig. 5e), expressing an increase more than 80%. The percentage of PAC-1-positive control platelets was 7.7% (Fig. 6b) and that of SP MS platelets 12.8% (Fig. 6e), so an increase was over 60%. In ADP-stimulated platelets, we observed the augmented level of PA (1.9-fold *vs*. control;* p* < 0.002) (Fig. 3b). This was confirmed by the representative dot plots of PA for healthy subjects (3.5%) (Fig. 7b) and SP MS patients (7.2%) (Fig. 7e). Similarly, we showed an increased pool of PMPs (2.3-fold *vs*. control; p < 0.002). The random combination of two representative dot plots of the percentage of PMPs for healthy control, i.e., 5.8% (Fig. 7b), and SP MS sample, i.e., 10.2% (Fig. 7e), gave a 1.8-fold increase of PMPs in SP MS.

**Fig. 4:**
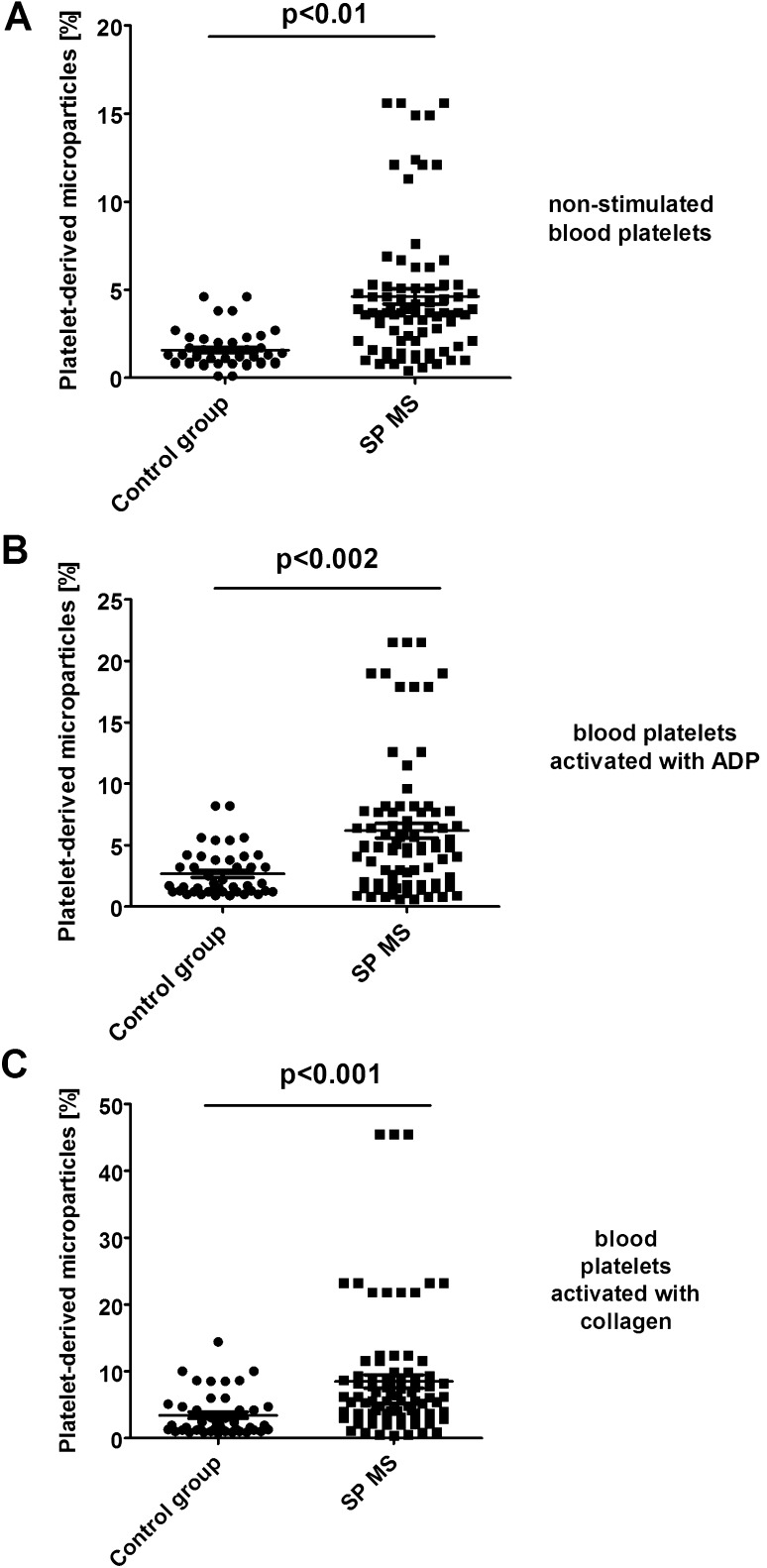
Formation of platelet-derived microparticles in control (**a**) and agonist-stimulated platelets: ADP 20 µM (**b**) and collagen 20 µg/ml (**c**) in whole blood samples obtained from SP MS patients and healthy controls. The fraction of platelet-derived microparticles was distinguished from platelets (CD61-positive objects) based on their size and granularity on the forward light scatter (FSC) *vs*. side light scatter (SSC) plots. CD61-positive objects with FSC lower than 10^2.3^ were characterized as PMPs. In each sample, 10 000 CD61-positive objects (platelets) were measured. Statistical analysis was performed using Mann–Whitney *U* test

**Fig. 5:**
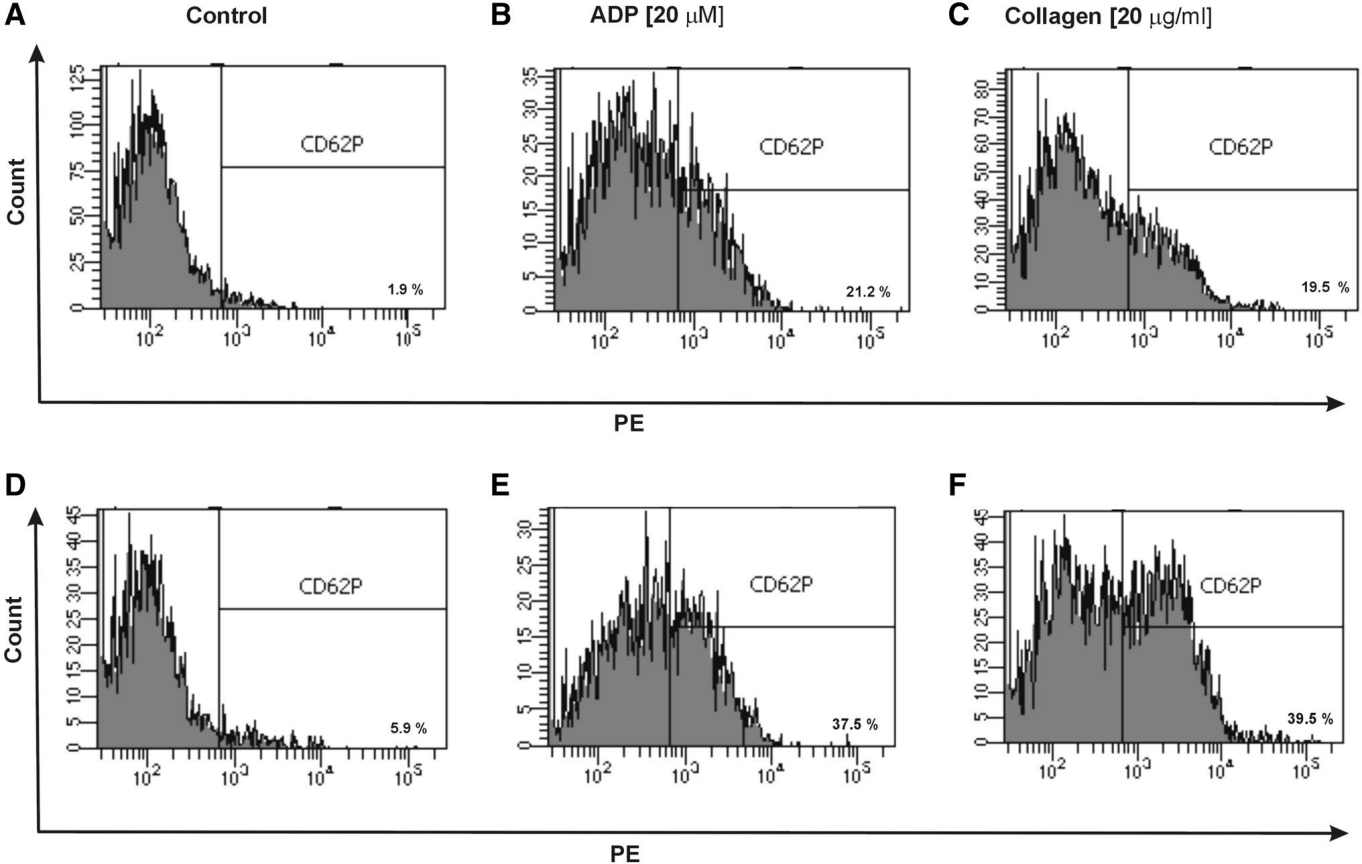
Representative histograms of the expression level of P-selectin on non-stimulated platelets (shown as control) (**a, d**) and on stimulated platelets with ADP (**b, e**) or collagen (**c, f**) in whole blood samples obtained from SP MS patients (**d**–**f**) and healthy controls (**a**–**c**). The blood platelets in whole blood were distinguished based on the expression of CD61 conjugated with PerCP. For each sample, 10 000 CD61-positive objects (platelets) were acquired. The blood platelets were labeled with monoclonal antibody anti-CD62P (PE)

**Fig. 6:**
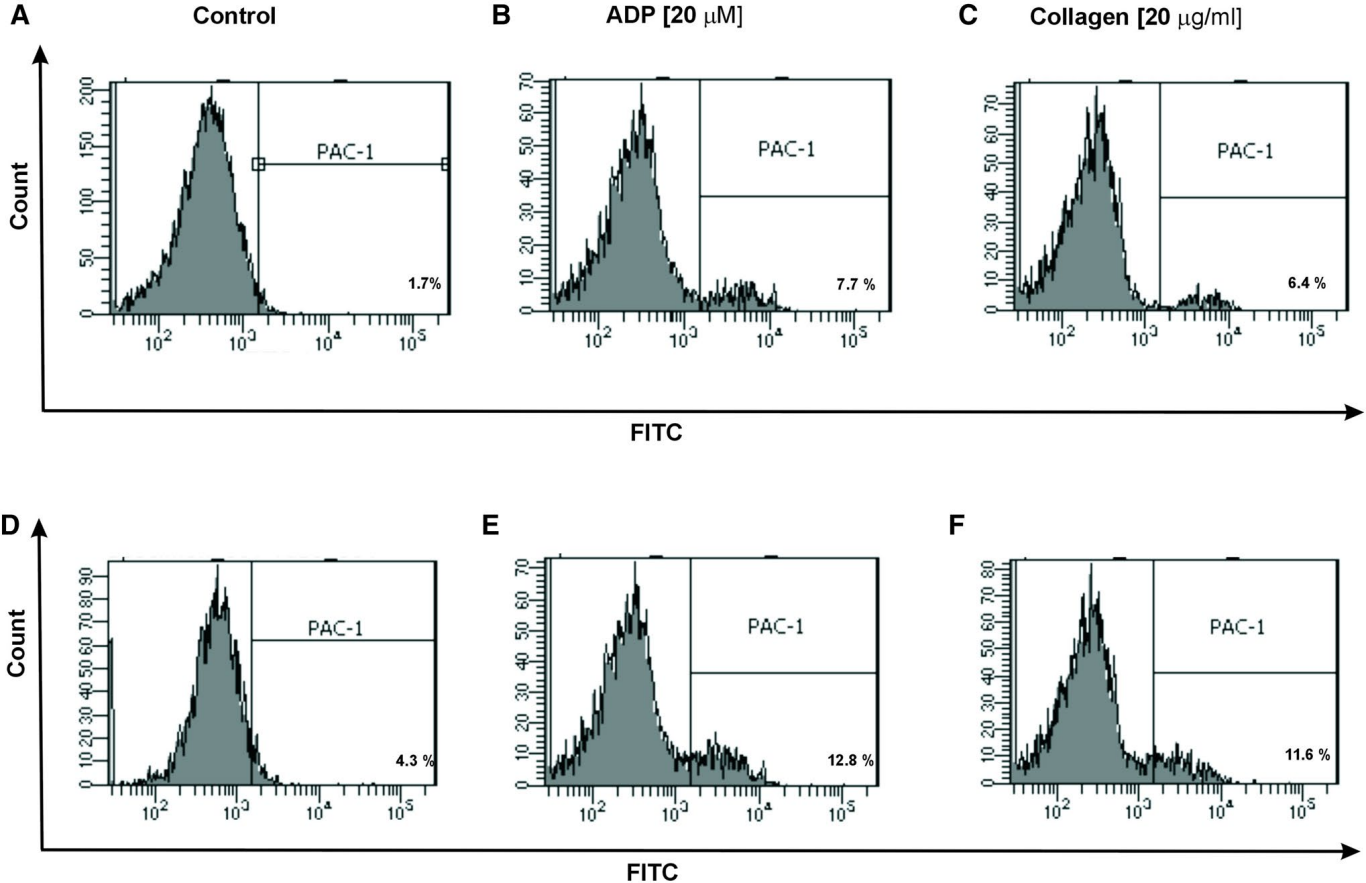
Representative histograms of the expression level of active form of GPIIb/IIIa on non-stimulated platelets (shown as control) (**a, d**) and on stimulated platelets with ADP (**b, e**) or collagen (**c, f**) in whole blood samples obtained from SP MS patients (**d**–**f**) and healthy controls (**a**–**c**). The blood platelets in whole blood were distinguished based on the expression of CD61 conjugated with PerCP. For each sample, 10 000 CD61-positive objects (platelets) were acquired. The blood platelets were labeled with monoclonal antibody PAC-1 (conjugated with FITC) against the active form of GPIIb/IIIa

**Fig. 7:**
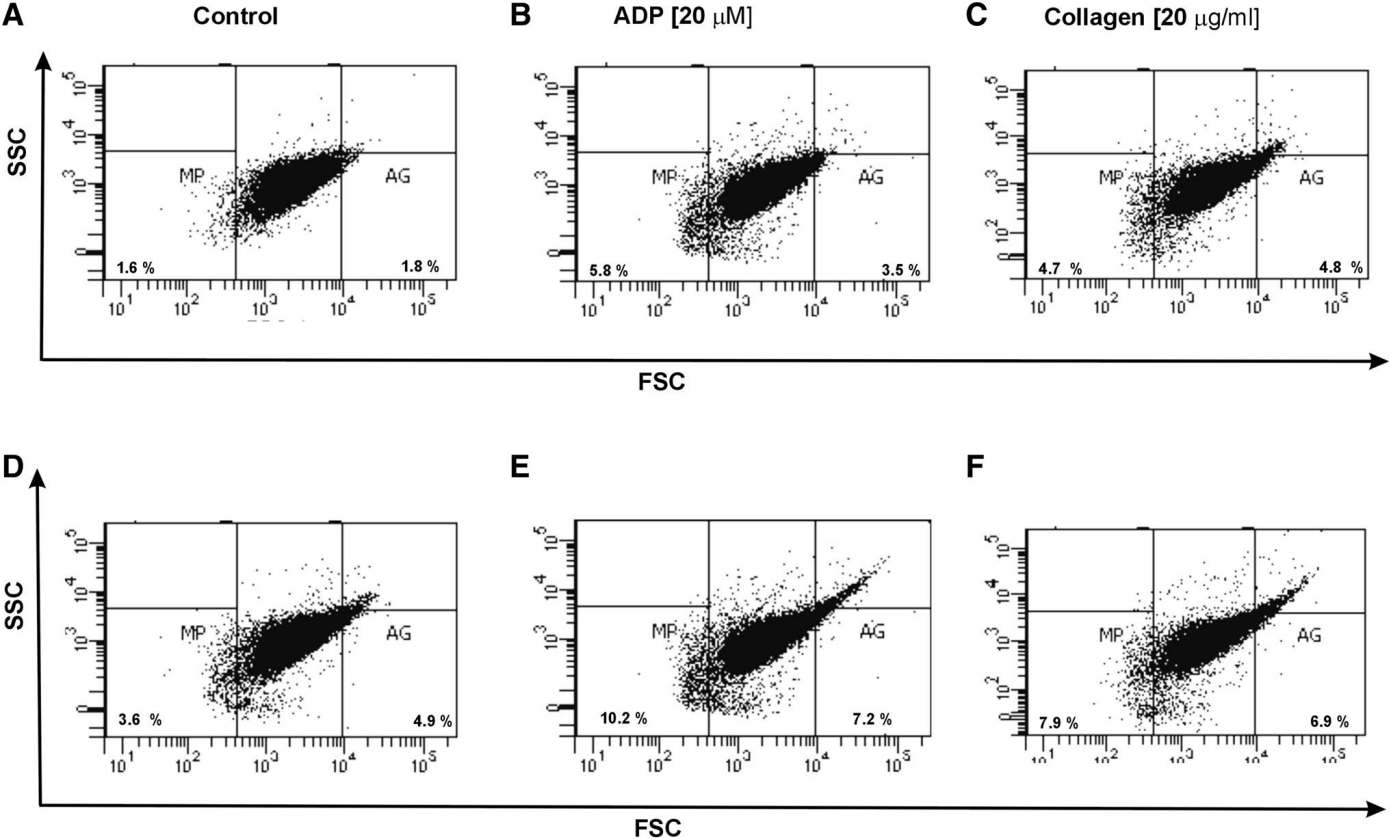
Representative dot plots of the percentage of platelet-derived microparticles and platelet aggregates in a population of resting platelets (shown as a control) (**a, d**) and in a population of stimulated platelets with ADP (**b, e**) or collagen (**c, f**) in whole blood samples obtained from SP MS patients (**d**–**f**) and healthy controls (**a**–**c**). The blood platelets in whole blood were distinguished based on the expression of CD61 conjugated with PerCP. For each sample, 10,000 CD61-positive objects (platelets) were acquired

The incubation of whole blood with 20 µg/ml of collagen also resulted in the statistically significant increase of blood platelet reactivity in SP MS patients compared to the control group. We noticed in SP MS group a twofold increase in CD62P expression (*p* < 0.001) (Fig. 1c), over a 1.9-fold increase in PAC-1 binding (*p* < 0.001) (Fig. 2c) and an increase in the formation of PA and PMPs: 2.1-fold (*p* < 0.05) (Fig. 3c) and 2.3-fold (*p* < 0.001) (Fig. 4c), respectively, vs. healthy subjects. The representative histograms of the expression level of P-selectin (Fig. 5) and the active form of GPIIb/IIIa (Fig. 6) on collagen-stimulated platelets from healthy subject (C) and SP MS patient (F) confirmed a twofold increase in the expression of CD62P and a 1.8-fold increase in PAC-1 binding. However, the representative dot plots of PMPs and PA for typical healthy control (C) and for selected patient (E) indicated a 1.4-fold increase in PMPs and a 1.7-fold increase in PA (Fig. 7).

**Fig. 8:**
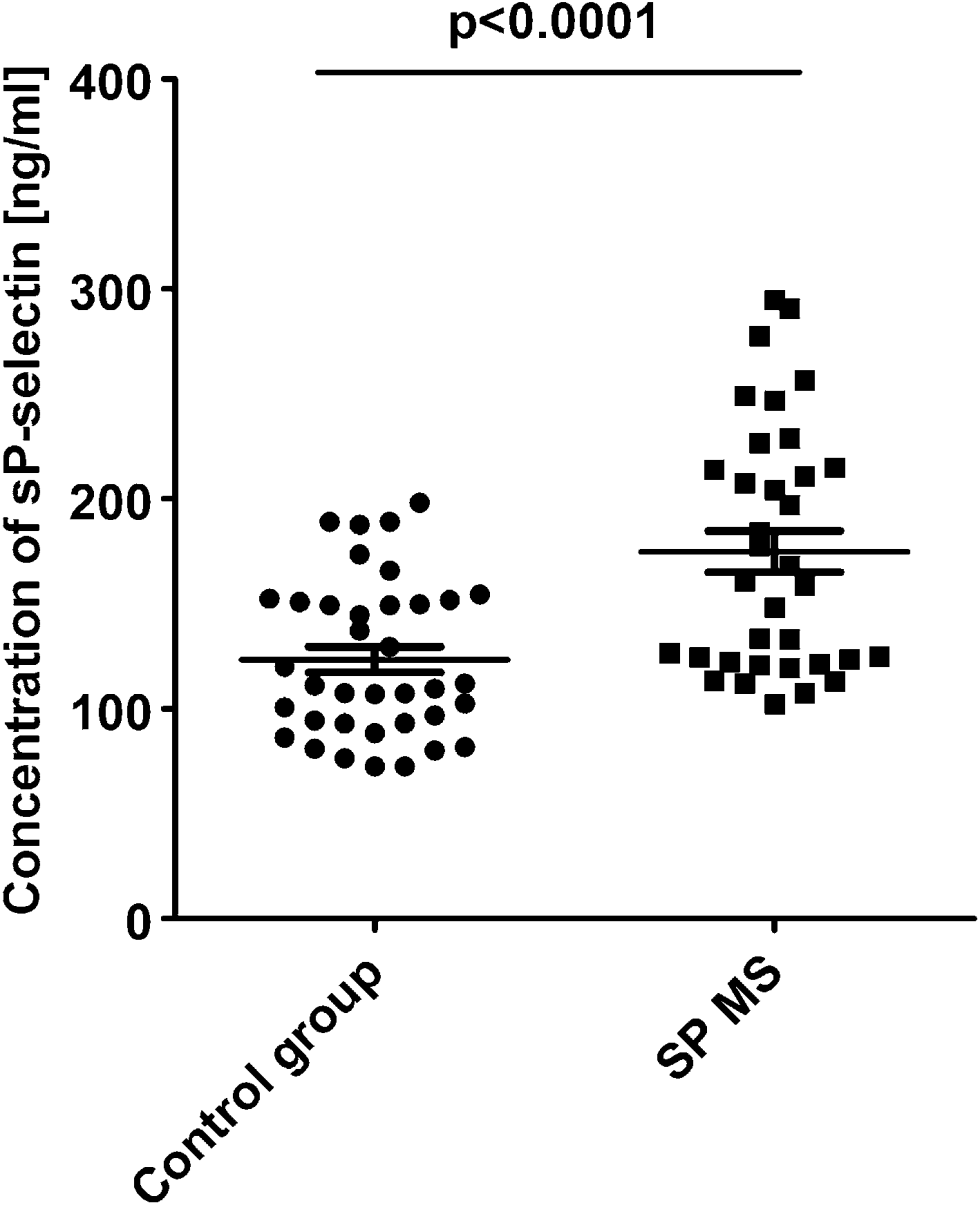
Level of sP-selectin in plasma obtained from SP MS patients and healthy controls. sP-selectin was determined by ELISA method. Statistical analysis was performed using unpaired Student’s *t* test (*p* < 0.0001)

Additionally, we compared the level of sP-selectin released into plasma. From the results obtained in ELISA test, we observed that the concentration of sP-selectin was statistically significantly higher (174 ± 8 ng/mL vs. 123 ± 6 ng/mL; *p* < 0.0001) in patients with SP MS than in healthy controls (Fig. 8).

## Discussion

Besides participation in hemostasis, blood platelets may be involved in the propagation and development of major diseases like neurodegenerative diseases, including MS [30]. Habets et al. examined the potential role of platelets in joint inflammation in rheumatoid arthritis (RA) and showed the increased expression of P-selectin, the elevated production of sCD40L, and the increase in platelet aggregation in patients with RA compared to healthy subjects. Moreover, these studies demonstrated the elevated plasma levels of TNF-α and PAF in RA, that is, the factors responsible for platelet activation through the arachidonic acid pathway. The authors demonstrated the positive correlation between activation of platelets and disease activity thus suggesting a role form platelets function in the course of autoimmune disease accompanied by inflammation [31].

Our previous studies clearly indicate that platelets isolated from patients with SP MS are more activated and are easily stimulated by exogenous stimuli, analogous to physiological agonist [27]. Our observations strongly support the results from earlier literature [32].

In the present work, for the first time, we assessed the degree of platelet activation in their natural environment, which is the whole blood analyzed immediately after blood collection. Blood platelets are readily activated and damaged during the procedure of isolation and purification. The possibility to apply flow cytometry to assess the platelets in whole blood without isolation of platelets or platelet-rich plasma significantly reduces the risk of creating artifacts. The measurement in whole blood using flow cytometric assay determines the activation state of circulating platelets, if it is done without the addition of external platelet agonists. Among the existing reports confirming the platelet abnormalities in MS patients, there are no studies of biological activity of platelets in whole blood using flow cytometry. The study of Sheremata et al. using flow cytometry method in PRP proved that blood platelets are significantly activated in RR MS patients [21]. In general, there are only very few data describing the physiology of platelets in the secondary progressive phase of MS. Roshanisef at et al. based on Poisson regression estimated the relative risk of cardiovascular disease in patients with MS: for RR MS reaching 2.16 and for SP MS reaching 3.4 [33]. They also showed that the risk of thromboembolic events associated with the procoagulant platelet activation increases with disease progression.

Among the microparticles from various types of cells, the most numerous are PMPs which represent up to 90% of all of them. They are the result of shedding of cell membranes and the formation of vesicles, so they are accepted markers of wear and disintegration of cells. An increased level of PMPs is typical of some diseases [34]. It is well known the important role of PMPs released from activated platelets, to catalyze the coagulation cascade. This is accomplished by an activation-dependent membrane inversion or “flip-flop” by which normally inward-facing phospholipids become exposed to plasma. These anionic phospholipids form procoagulant surface by promoting coagulation factors into their active complexes, leading to the generation of thrombin that converts fibrinogen to the fibrin plug [23]. Some epidemiological studies confirm that MS increases the risk of cardiovascular events, mainly venous thromboembolism, ischemic strokes, and myocardial infarction [35]. Moreover, PMPs reveal most of the platelet membrane proteins, including the matrix metalloproteinases which have been generally recognized as major participants in the disruption of BBB in MS, by enabling the migration of white blood cells to the CNS [16]. As has been shown, PMPs may play an important role in neurodegenerative diseases and their increased expression is an accepted marker of platelet activation [36, 37]. Our findings in patients with SP MS are in line with the results obtained by Sheremata et al., which indicated the elevated levels of PMPs in RR MS patients [21]. However, thanks to the analysis conducted in whole blood, we could, for the first time, successfully confirm the speculation presented in the earlier literature that platelets are chronically activated in MS. Flow cytometry method allows for comparative analysis in study and control samples, not only a fraction of PMPs, but also the pool of aggregates, as objects larger than a single cell. We demonstrated the increased percentage of platelet–platelet and/or platelet–leukocyte aggregates presented in whole blood of patients with SP MS, relative to healthy controls (Fig. 3a).

For the analysis of platelet surface marker expression on the surface of platelets in non-stimulated whole blood, the number of associated monoclonal antibodies, expressed as the fraction of antigen-positive platelets relating to some glycoproteins platelet membrane (e.g., P-selectin, GPIIb/IIIa) is a marker for platelet activation in circulation [38]. The presence and quantification of the active complex of glycoproteins IIb and IIIa (acting as the main receptor for fibrinogen) on the platelet surface depend on the state of platelet activation. GP IIb/IIIa belongs to the constituent antigens, which are always present on the cell surface, including non-stimulated platelets, but their number has been growing significantly as a result of platelet activation, due to the release of contents from intracellular granules [39]. The conformational changes in GPIIb/IIIa complex upon platelet activation lead to binding of fibrinogen and von Willebrand factor (vWF), and, in consequence, to platelet aggregation [40, 41].

Previous reports have shown the presence of platelets in the inflamed spinal cord tissue in the course of murine experimental autoimmune encephalomyelitis (EAE) [18], as well as in human MS lesions [42]. Moreover, microarray analysis of differentially expressed genes in MS plaques obtained at autopsy proved an upregulation of the genes for platelet-specific GPIIb in patients with chronic demyelinating disease [42]. Similar results were obtained in a murine model of EAE. As compared to non-inflamed spinal cords, the elevated level of GPIIb mRNA was found in the inflamed spinal cords of mice on day 21 after EAE induction using myelin oligodendrocyte glycoprotein. Moreover, the authors suggested that the contribution of platelets to EAE is probably associated with the activation of GPIIb/IIIa complex, because blockade of this receptor reduced EAE severity in mice [18]. Our results are in line with the mentioned reports. We have shown an increased surface expression of GPIIb/IIIa active complex on platelets in whole blood of patients with SP MS. A significant increase was about 55% compared to healthy controls (Fig. 2a).

One of the main biomarkers of platelet activation is P-selectin, a glycoprotein presented in α-granules of resting platelets. The activated platelets dynamically change their shape and release the contents of α-granules, secreting a variety of cytokines, chemokines, and growth factors. During platelet activation, CD62P appears on their surface and allows platelet–platelet or platelet–leukocyte interaction *via* P-selectin glycoprotein ligand-1 (PSGL-1) presented on leukocytes’ surface (mainly on monocytes and neutrophils). The formation of platelet–leukocyte aggregates leads to the production of inflammatory molecules, such as leukotrienes and cytokines, and augments the inflammation. Platelet–leukocyte interaction may occur as a consequence of platelet activation and possibly plays a key role in the deposition of activated platelets in demyelinating lesions in MS, which leads to brain neurodegeneration [21, 43].

The expression of CD62P on the platelet surface reflects cell degranulation, and therefore the measurement of its level is a useful tool to monitor the activation status of platelets in vitro and in vivo [44, 45]. Based on flow cytometric measurements, we have shown a significantly higher (about 115%) basal P-selectin expression on the platelet surface in SP MS patients in comparison to the control group (Fig. 1). Our findings are similar to the results obtained by Sheremata et al., who demonstrated significantly elevated expression of CD62P in RR MS [21]. Sheremata et al. using flow cytometric method demonstrated that blood platelets were significantly activated in patients with RR MS. The authors, for the first time, observed that the levels of accepted markers of platelet activation, PMPs and CD62, were, respectively, over twofold and 1.7-fold higher in patients with RR MS in comparison to healthy controls [21]. We noticed the slightly higher platelet activation in patients with SP MS: the pool of PMPs (2.5-fold vs. control) and the expression of CD62 (2.3-fold *vs*. control) were elevated. Both in our study and that of Sheremata et al., the level of circulating platelet microaggregates was extended.

CD62 belongs to antigens that are temporarily expressed on the platelet surface upon activation and over time undergo proteolytic cleavage into plasma. Platelets are the major source of circulating soluble P-selectin. Detection of elevated level of this antigen in plasma proves that the previous episodes of platelet activation took place in circulation [28]. The elevated plasma level of sP-selectin is a biomarker of vascular disease and is also associated with several inflammatory disorders. Soluble P-selectin has functional effects by activating leukocytes and promoting their adhesion and subsequent recruitment to arterial and venous micro- and macrocirculation [43, 46]. The increased expression of sP-selectin level was observed in the murine model of MS [47]. Kuenz et al. also investigated platelet activation by the measurement of sP-selectin plasma concentration in MS patients. The authors observed a higher level of sP-selectin in RR MS compared to the group of healthy blood donors [43]. In our research, we noticed not only the raised level of P-selectin membrane expression, but also the increased plasma level of sP-selectin (over 41%) in SP MS patients in comparison to healthy subjects (Fig. 8). Our results argue in favor of the high frequency of episodes of platelet activation in the circulation of patients with SP MS. This is evidenced by the increased level of P-selectin detected both on the platelet surface and in plasma.

With the additional application of exogenous activating factors (identical to physiological) into the samples of whole blood, cytometry method allows to determine the reactivity of platelets ex vivo in their physiological environment. Platelet response to the physiological agonists is a measure of the availability and depletion of circulating platelets. To determine the functionality of platelets in SP MS, we measured the levels of activation markers after stimulation with ADP or collagen. In this study, we showed the enhanced reactivity of platelets in whole blood of patients with SP MS. By measuring the expression levels of P-selectin (Figs. 1b, c, 4) and GPIIb/IIIa (Figs. 2b, c, 5) and determining the fraction of circulating PA (Figs. 3b, c, 6) and PMPs (Figs. 4b, c, 6), we showed that platelets of patients with SP MS were fully functional, but exhibited an increased response to the agonists, characteristic of chronic activation.

## Conclusions

In the present work, for the first time, we demonstrated the significantly higher platelet activation in SP MS patients in their natural environment, which is the whole blood analyzed immediately after collection. Therefore, we can conclude that the disease progression of SP MS is associated with platelet hyperactivity. Furthermore, augmented levels of platelet aggregation and generation of PMPs can be the main causes of the increased risk of thromboembolic events, which are observed in patients with SP MS. Moreover, as we have shown platelets from patients with SP MS are more sensitive to agonists and are more easily activated. We can therefore assume that platelets, which are involved in the development of inflammatory and autoimmune responses, as well as disorders of hemostasis, are one of the key targets of therapy in SP MS. The understanding of molecular processes of platelet function underlying the SP MS might be beneficial and may improve more specific treatments of SP MS in the future.
